# Value of CT Perfusion for Collateral Status Assessment in Patients with Acute Ischemic Stroke

**DOI:** 10.3390/diagnostics12123014

**Published:** 2022-12-01

**Authors:** Haryadi Prasetya, Manon L. Tolhuisen, Miou S. Koopman, Manon Kappelhof, Frederick J. A. Meijer, Lonneke S. F. Yo, Geert J. Lycklama á Nijeholt, Wim H. van Zwam, Aad van der Lugt, Yvo B. W. E. M. Roos, Charles B. L. M. Majoie, Ed T. van Bavel, Henk A. Marquering, on behalf of the MR CLEAN Registry Investigators

**Affiliations:** 1 Department of Biomedical Engineering and Physics, Amsterdam UMC, Location AMC, 1105 AZ Amsterdam, The Netherlands; 2 Department of Radiology and Nuclear Medicine, Amsterdam UMC, Location AMC, 1105 AZ Amsterdam, The Netherlands; 3 Department of Radiology, Radboud UMC, 6525 GA Nijmegen, The Netherlands; 4 Department of Radiology, Catharina Hospital, 5623 EJ Eindhoven, The Netherlands; 5 Department of Radiology, Haaglanden Medical Center, 2512 VA The Hague, The Netherlands; 6 Department of Radiology, Maastricht University Medical Center and Cardiovascular Research Institute, 6229 ER Maastricht, The Netherlands; 7 Department of Radiology and Nuclear Medicine, Erasmus MC University Medical Center, 3015 GD Rotterdam, The Netherlands; 8 Department of Neurology, Amsterdam UMC, Location AMC, 1105 AZ Amsterdam, The Netherlands

**Keywords:** perfusion, CTP, collaterals, ischemic stroke

## Abstract

Good collateral status in acute ischemic stroke patients is an important indicator for good outcomes. Perfusion imaging potentially allows for the simultaneous assessment of local perfusion and collateral status. We combined multiple CTP parameters to evaluate a CTP-based collateral score. We included 85 patients with a baseline CTP and single-phase CTA images from the MR CLEAN Registry. We evaluated patients’ CTP parameters, including relative CBVs and tissue volumes with several time-to-maximum ranges, to be candidates for a CTP-based collateral score. The score candidate with the strongest association with CTA-based collateral score and a 90-day mRS was included for further analyses. We assessed the association of the CTP-based collateral score with the functional outcome (mRS 0–2) by analyzing three regression models: baseline prognostic factors (model 1), model 1 including the CTA-based collateral score (model 2), and model 1 including the CTP-based collateral score (model 3). The model performance was evaluated using C-statistic. Among the CTP-based collateral score candidates, relative CBVs with a time-to-maximum of 6–10 s showed a significant association with CTA-based collateral scores (*p* = 0.02) and mRS (*p* = 0.05) and was therefore selected for further analysis. Model 3 most accurately predicted favorable outcomes (C-statistic = 0.86, 95% CI: 0.77–0.94) although differences between regression models were not statistically significant. We introduced a CTP-based collateral score, which is significantly associated with functional outcome and may serve as an alternative collateral measure in settings where MR imaging is not feasible.

## 1. Introduction

In patients with acute ischemic stroke, leptomeningeal collateral blood flow potentially maintains blood supply to the ischemic region until the occluded vessel is revascularized [1]. Good collateral status is associated with favorable outcomes, smaller infarct volumes, and lower incidences of hemorrhagic transformation following endovascular therapy [2,3,4,5,6,7,8,9,10].

Collateral capacity can be assessed using several imaging modalities, including DSA, CTA, and MRA [11,12,13,14]. MRA and CTA have been used to indirectly assess collateral status based on contrast filling in the arteries distal to the clot. In those studies, the collateral status is graded by classifying the extent, the intensity, the speed, or combinations of these contrast filling variables in arteries downstream of the thrombus.

These collateral grading systems have coarse qualitative grading scales and their own limitations. For example, CTA is sensitive to inaccurate scan timing and may miss slower retrograde contrast enhancement of the pial arteries because of the lack of temporal resolution [6]. Collateral grading based on (single vessel) DSA allows only a limited assessment of the MCA territory and is only available after a patient has been selected for treatment [15]. Although these approaches provide an indication of collateral capacity, they do not offer information on the local perfusion of the affected tissue. Perfusion-based imaging acquisitions may provide improved estimates of collateral status in addition to their value in the assessment of stroke pathophysiology and penumbra volume [16].

A recent study suggested that MR perfusion allows for the quantitative assessment of collateral status with a high agreement with a DSA-based collateral score [17]. In that study, perfusion parameters, such as the time delay of the tissue residue function and the corresponding blood volume, were combined to determine a perfusion collateral index. Because the arterial time delay and relative CBV (rCBV) are generated automatically by MR perfusion imaging software, the perfusion collateral index can be calculated quickly and independent of expert readers. In the time-critical setting of acute stroke care, such rapid assessment of collateral status may provide added clinical value and factor into therapeutic decision-making.

Compared to CT, MRI has a number of limitations in the acute setting, including its limited availability and longer acquisition times [18]. CT is more widely available in acute stroke care settings. Moreover, CTP is increasingly performed in clinical practice. We hypothesize that, next to MR perfusion, CTP also allows for the assessment of the collateral capacity. We aimed to evaluate various baseline CTP parameters to select a CTP-based collateral score (CTP-CS). We subsequently aimed to assess the association of this CTP-CS with functional outcome after endovascular treatment for acute ischemic stroke.

## 2. Materials and Methods

### 2.1. Patients

The MR CLEAN Registry (A Multicenter Clinical Registry of Endovascular Treatment for Acute Ischemic Stroke in the Netherlands) is a prospective, multicenter registry collecting data of patients treated with endovascular treatment for ischemic stroke from all stroke intervention centers in the Netherlands. In this study, we selected patients from the MR CLEAN Registry who were treated between June 2016 and November 2017 and for whom baseline CTP and CTA data were available. We further included patients with an occlusion of the M1- or M2-segment of the middle cerebral artery. We excluded patients with poor scan quality due to motion artefact, insufficient contrast or noise, and low temporal imaging resolution. Collateral scores based on baseline single-phase CTA images (CTA-CS) and functional outcomes at 90 days (assessed with mRS) were collected [19].

### 2.2. CT Perfusion Analysis

CTP data were analyzed using a commercially available software package (Syngo.via; Siemens Healthineers, Erlangen, Germany) to generate perfusion parameters, i.e., CBF, CBV, MTT, and the time to the maximum of residue function (Tmax). For each dataset, the software automatically stripped the skull by finding the bone contour and removed both cerebrospinal fluid and calcifications by intensity thresholding. The locations to assess the global arterial input function and venous output function were automatically determined at the internal carotid artery and superior sagittal sinus, respectively. Thresholding was performed to remove peripheral and perforating vessels, as their inclusion could lead to the overestimation of microvascular flow. Subsequently, the software generated time attenuation curves of contrast enhancement in Hounsfield Units at the arterial input function and venous output function locations, and in each voxel in the brain area. CBV, CBF, MTT, and Tmax for every voxel of brain tissue were then calculated from the time attenuation curve-derived residue function [20].

A moderately hypoperfused area is indicative of penumbra, which is sustained by collaterals [21,22]. We chose two CTP parameters to represent the delay and dispersion components of collaterals in moderately hypoperfused areas: Tmax and rCBV [17]. rCBV is defined as the volume of intravascular blood in mL per 100 mL of the brain, compared to that in the contralateral hemisphere.

### 2.3. Additional Imaging Assessment

**Figure 1:**
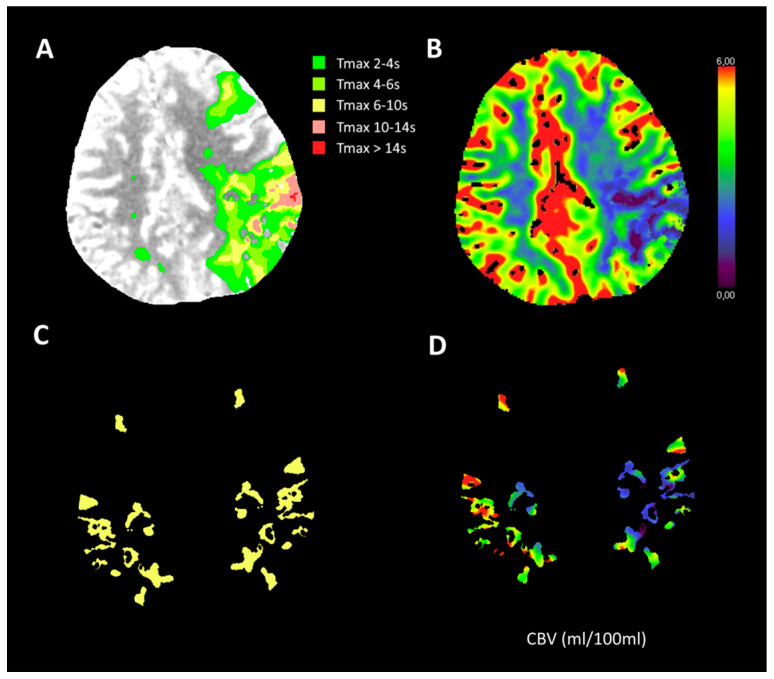
Illustration of one measure as a candidate for baseline CTP-based collateral score: relative CBV multiplied by the total volume of brain tissue defined by time-to-maximum (Tmax). (**A**): Areas with various Tmax value ranges; (**B**): The corresponding CBV values of A (mL/100 mL); (**C**): Mask for Tmax 6–10 s. The contralateral mask is acquired by mirroring the ipsilateral mask using the midline; (**D**): CBV within mask C. The score is the mean CBV of the ipsilateral masked volume relative to the mean CBV of the contralateral masked volume multiplied by the total volume of all the voxels with a Tmax value of 6–10 s.

We chose two measures each for six different ranges of Tmax as candidates for CTP-CS. The two measures were the mean rCBV of the volume defined by Tmax (rCBV_Tmax(t1)–(t2)_) and this rCBV multiplied by the total volume of brain tissue as defined by the Tmax (Vol_Tmax(t1)–(t2)_). To calculate the measures, we first created Tmax masks, which included all voxels within the predefined Tmax ranges. The contralateral mask was created by mirroring the ipsilateral mask in the midplane. rCBV was calculated as the mean CBV of the ipsilateral masked volume divided by the mean CBV of the contralateral masked volume. The total volume of grey and white matter was calculated by multiplying the voxel volume with the number of voxels that had Tmax values within the given range. The six predefined ranges of Tmax, which depicted the different degrees of hypoperfusion, were 2–4 s, 4–6 s, 2–6 s, 6–10 s, 4–10 s, and 10–14 s. In total we evaluated 12 CTP-CS candidates and subsequently selected one measure as the CTP-CS for further analysis. Figure 1 shows how the rCBV multiplied by the Tmax-based tissue volume was calculated. An example for the mask of Tmax 6–10 s is also shown.

We used data on CTA occlusion locations and collateral scores assessed by an independent core laboratory of neuroradiologists [19]. CTA-CS was based on a 4-point scale: 0 for absent collaterals (no filling of the territory distal to the occlusion), 1 for poor collaterals (less than half filling of the territory), 2 for moderate collaterals (more than half filling of the territory), and 3 for good collaterals (complete filling of the territory) [23]. The unaffected contralateral hemisphere was used as a reference to evaluate the contrast filling.

### 2.4. Statistical Analysis

Continuous and categorical variables were summarized as median (interquartile range, IQR) and frequency (percentage), respectively. We used the Jonckheere-Terpstra test to determine which CTP-CS candidate had a significant association with CTA-CS and a 90-day mRS. The associations of CTA-CS and the optimal CTP-CS measure with functional independence (mRS 0–2) were assessed using multivariable logistic regression models. For this analysis, we evaluated three models. In the base model (model 1), baseline prognostic factors including age, NIHSS, time from onset to groin puncture, history of hypertension, diabetes mellitus, and previous strokes were included. In model 2, we added the CTA-CS to model 1. In model 3, the CTP-CS was added to model 1. The adjusted OR for statistically significant predictors were reported with 95% CI to indicate statistical precision. Receivers operating characteristics were subsequently determined to compare the predictive power of the models in distinguishing favorable from unfavorable functional outcomes. We compared the C-statistics between models using likelihood ratio tests. We used the Akaike information criterion to compare the relative quality of the regression models. Lower Akaike information criterion implies a more parsimonious model. *p*-values smaller than 0.05 were considered statistically significant. All statistics were performed using IBM SPSS software (version 19.0.0).

## 3. Results

**Figure 2:**
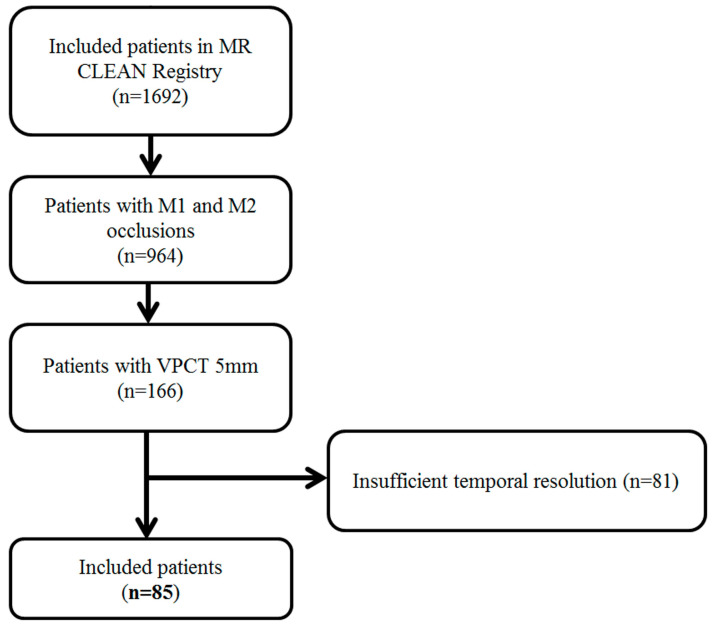
Flowchart of patient inclusion in the study.

A total of 85 patients were included in our analysis (Figure 2). Table 1 shows the baseline characteristics of the patients included in the study. The median age of the patients was 75 years (IQR 63–81); 41 patients (48%) were female, and the median NIHSS was 16 (IQR 11–20). Core-lab determined that the CTA collateral score was 0 in 4 patients (5%), 1 in 37 patients (43%), 2 in 37 patients (43%), and 3 in 7 patients (9%).

The Jonckheere-Terpstra test showed that among the 12 candidates we evaluated, only the mean rCBV of the Tmax between the 6 and 10 s range (rCBV_Tmax6–10_) was significantly associated with the change of both CTA-CS and ordinal mRS (Table 2). We therefore selected rCBV_Tmax6–10_ as CTP-CS for further analysis.

**Figure 3:**
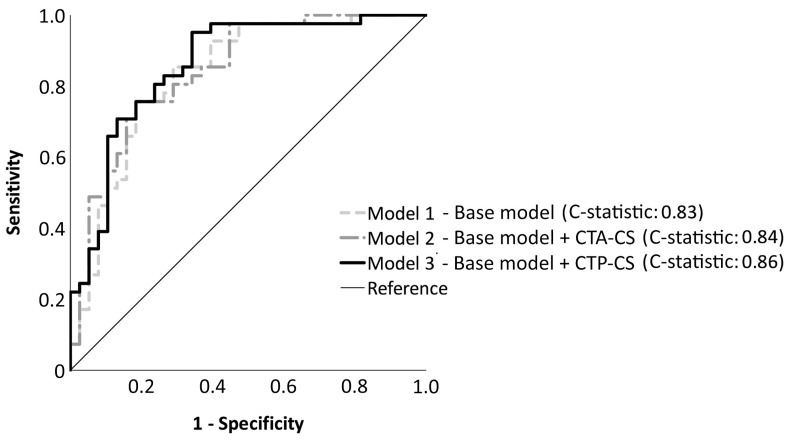
Receiver-operating characteristics curves for three different models for predicting favorable functional outcomes (mRS 0–2). Model 1 is the base model with baseline prognostic factors as the independent variables: age, stroke severity (NIHSS), time from onset to groin puncture, history of hypertension, diabetes mellitus, and previous strokes. Model 2 and model 3 are model 1 with the addition of collateral scores as assessed by CTA and CTP, respectively. The C-statistic for model 1, model 2, and model 3 respectively are 0.83 (95% CI, 0.74–0.92), 0.84 (95% CI, 0.75–0.93), and 0.86 (95% CI, 0.77–0.94). There are no significant differences between the C-statistics of the models.

Six patients with missing outcome variables were excluded from the multivariable regression analyses. We found that in our patient population, CTA-CS was not significantly associated with a favorable outcome (*p* = 0.26), as shown in Table 3. On the other hand, CTP-CS was significantly associated with favorable outcomes with adjusted OR 1.04 (95% CI, 1.002–1.068, *p* = 0.036) per 1% increase of CTP-CS. Regression model analysis showed that the C-statistic for model 1 was 0.83 (95% CI, 0.74–0.92; Table 4). With the addition of CTA-CS, model 2 had a C-statistic of 0.84 (95% CI, 0.75–0.93). Finally, favorable outcomes were most accurately predicted by model 3 with a C-statistic of 0.86 (95% CI, 0.77–0.94). This model also had the lowest Akaike information criterion. The differences between the C-statistics of the regression models were not statistically significant (model 1 vs. model 2, *p* = 0.88; model 2 vs. model 3, *p* = 0.75; model 1 vs. model 3, *p* = 0.63). Figure 3 shows the receiver operating characteristics curves of the three regression models.

## 4. Discussion

We showed that the CTP parameters Tmax and rCBV can be used to automatically assess collateral capacity in patients who received endovascular treatment for acute ischemic stroke due to a proximal anterior circulation occlusion.

We consider the rCBV of moderately hypoperfused volumes to be a proxy for collaterals; it is an estimate of how much a collateralized microvascular volume is reduced from its healthy volume. The hypoperfused volume represents penumbra which is likely sustained by collaterals [24,25,26,27]. Therefore, the delayed perfusion time may be indicative of collateral status [28]. This was confirmed in multiple MR perfusion studies that associated delayed perfusion time with collateral status [17,28,29,30,31,32]. In addition to the delay, the microvascular blood volume of the hypoperfused area may be indicative of the dispersion of collateral flow [33,34,35]. Delay and dispersion are two important features for the accurate determination of collateral status [34]. For example, the late arrival time (delay), the speed of vessel filling, and the amount of contrast (dispersion) in pial arteries provide insights into leptomeningeal collateral status [36]. The use of rCBV as CS-CTP relies on having a substantial amount of moderately hypoperfused volume at the time of measurement. This constraint avoids possible mirroring errors during contralateral mask creation. Additionally, sufficient volume suppresses noise which otherwise could negatively impact the reading accuracy. Consequently, the CTP-CS may not be suitable for patients with an insufficient penumbra volume.

We expected that blood volume would be lower on the affected side compared with the unaffected side, resulting in an rCBV ranging from 0 to 1, with a higher rCBV indicating better collaterals [35,37]. Interestingly, this did not seem to be the case as most rCBV medians were larger than 1. One possible explanation is the loss of vascular tone and the recruitment of capillaries in the penumbral microcirculation as a response to hypoxia [38].

A study on MR perfusion-based collateral assessment suggests that an arterial time delay of 2–6 s best describes the moderately hypoperfused volume [17]. The follow-up study with CTP confirmed that an arterial time delay of 2–6 s was indicative of collateralization [39]. We could not reproduce this finding in our CTP-based study as the volume of brain tissue with a Tmax of 2–6 s, although showing the expected trend, was not significantly associated with either the CTA collateral score nor the ordinal mRS in our patient population. This discrepancy may stem from a bias selection in the population and the inherent differences between the hemodynamical parameters estimation methods employed between their study and ours: the arterial time delay parameter is a Bayesian-estimated Tmax as opposed to the Tmax derived from a singular value decomposition model [40]. The 6 s threshold for determining hypoperfused tissue is consistent with other studies which use MR perfusion-weighted imaging on the DEFUSE study population [29,31,41]. The volume of this hypoperfused tissue had been used to categorize the collateral extent in the infarcted hemisphere [31,42]. The hypoperfusion intensity ratio, the volume of the tissue with a Tmax > 10 s divided by the volume of the tissue with a Tmax > 6 s, has shown to be significantly associated with a persistent perfusion profile for more than 38 h which may indicate favorable collaterals [43]. A recent study on the CTP-based hypoperfusion intensity ratio of 22 patients demonstrated significant associations between the ratio, using Tmax > 10 s and Tmax> 4 s, and a dynamic CTA-based collateral score and functional outcomes [44]. The evaluation of such thresholds in our collateral formulation did not show an association between the CTA collateral score and the functional outcome.

Most collateral grading methods require extensive assessment from experienced neuroradiologists, which might introduce bias [45]. Inter-observer agreements for collateral grading in various imaging modalities were insufficiently investigated, although some studies reported fair to good agreements [36,46]. The observer dependency is alleviated in our method because the entire process, from reading the CTP source images to generating the collateral score, is fully automated.

We acknowledge several limitations in this study. We included only cases with a middle cerebral artery occlusion. The CTP-based collateral score could be generalized to more proximal or distal occlusion cases, although the perfusion characteristics of the tissue sustained by different types of collaterals may not share the same properties with the tissue supplied by leptomeningeal collaterals. Furthermore, the high threshold of Tmax for our CTP-CS may exclude benign oligemia, which is within the domain of collaterals. We did not investigate the potential contributions of slow flow from pervious thrombus or incomplete occlusions into the moderately hypoperfused tissue, thus confounding the collateral assessment. We also recognize that a considerable amount of penumbra volume is necessary to ensure that the signal to noise ratio is large enough to limit an inaccurate estimation of collateral capacity. Moreover, we found no significant association between CTA-based collateral scores and functional outcomes [47]. This may be caused by imbalanced data due to the lack of samples in extreme grades, i.e., 4 patients with CTA-CS 0 and 9 patients of CTA-CS 3. In addition, some difficult cases in intermediate grades may have complicated the reading. Finally, the prominent discrepancy between CTP software packages may require a fine-tuning of the perfusion parameters to achieve similar results [48,49]. Further studies are warranted to evaluate the robustness of the CTP-based collateral score on different patient populations with a proximal anterior circulation occlusion.

## 5. Conclusions

This study demonstrates that the CTP parameters Tmax and rCBV can be used to automatically assess collateral capacity in patients who underwent endovascular treatment for acute ischemic stroke due to a proximal anterior circulation occlusion. We selected the mean of relative CBVs of the area with a Tmax of 6–10 s as the CTP collateral score because of its associations with both CTA collateral score and functional outcome. In addition, we showed that the multivariable prognostic model with the CTP-collateral score outperforms models without a collateral score or with the CTA-based collateral score, although these differences were not statistically significant. Because the perfusion parameters are automatically generated by CTP software, CTP-CS is quickly available and does not require an expert reader, potentially increasing its clinical utility in acute stroke settings.

## Figures and Tables

**Table 1:** Baseline Characteristics.

*N*	85
Age, median (IQR)	75 (63–81)
Female sex	41 (48%)
M1 occlusion of CT	66 (78%)
NIHSS, median (IQR)	16 (11–20)
History of ischemic stroke	18 (21%)
History of hypertension	42 (50%)
History of diabetes melitus	8 (9%)
Prestroke mRS	
0	60 (70%)
1	11 (13%)
≥2	14 (17%)
RR systolic in mmHg, median (IQR)	144 (130–160)
Treatment with IV-rtPA	62 (73%)
ASPECTS, median (IQR)	9 (9–10)
CTA collateral score	
0	4 (15%)
1	37 (43%)
2	37 (43%)
3	7 (9%)
eTICI	
0	13 (15%)
1	4 (5%)
2A	16 (19%)
2B	14 (16%)
2C	13 (15%)
3	25 (30%)
General anesthesia	7 (8%)
Onset-to-groin puncture time in min, median (IQR)	150 (118–211)
EVT time in min, median (IQR)	52 (31–81)

IQR indicates interquartile range; M1, M1 segment of middle cerebral artery; eTICI, extended treatment in cerebral ischemia; and EVT, endovascular treatment.

**Table 2:** Results of Jonckheere-Terpstra tests of the associations of the baseline CTP-based collateral score (CTP-CS) candidates with baseline single-phase CTA-based collateral score (CTA-CS) and ordinal 90-day mRS.

ECTP-CS Candidates	*p*-Value	
	Association with CTA-CS	Association with mRS
rCBV_Tmax2–4_ × Vol_Tmax2–4_ (mL)	0.028 ^a^	0.19
rCBV_Tmax4–6_ × Vol_Tmax4–6_ (mL)	0.99	0.91
rCBV_Tmax6–10_ × Vol_Tmax6–10_ (mL)	0.036 ^a^	0.35
rCBV_Tmax2–6_ × Vol_Tmax2–6_ (mL)	0.13	0.39
rCBV_Tmax4–10_ × Vol_Tmax4–10_ (mL)	0.16	0.56
rCBV_Tmax10–14_ × Vol_Tmax10–14_ (mL)	<0.001 ^a^	0.12
rCBV_Tmax2–4_	0.18	0.89
rCBV_Tmax4–6_	0.049 ^a^	0.59
rCBV_Tmax6–10_	0.020 ^a^	0.045 ^b^
rCBV_Tmax2–6_	0.16	0.80
rCBV_Tmax4–10_	0.038 ^a^	0.23
rCBV_Tmax10–14_	0.036 ^a^	0.09

Tmax: time-to-maximum of residue function; Vol_Tmaxa–b_: tissue volume as indicated by Tmax *a*–*b* s; rCBV_Tmaxa–b_: mean relative CBV of the brain tissue as indicated by Tmax *a*–*b* s. ^a^ Significantly different between groups defined by CTA collateral score (*p* < 0.05). ^b^ Significantly different between groups defined by mRS (*p* < 0.05).

**Table 3:** Adjusted Odds Ratio of logistic regression models of CTA-CS and CTP-CS for favorable outcome.

	Adjusted OR	95% CI	*p*-Value
CTA-CS per grade	1.62	0.70–3.73	0.26
CTP-CS per 1%	1.04	1.002–1.068	0.036

OR: odds ratio; CI: confidence interval; CTA-CS: baseline single phase CTA-based collateral score; CTP-CS: baseline CTP-based collateral score.

**Table 4:** Logistic regression models for favorable functional outcome with C-statistics and Akaike information criterion.

Model	C-Statistic (95% CI)	AIC
Model 1—Baseline prognostic factors	0.83 (0.74–0.92)	93.5
Model 2—Baseline prognostic factors + CTA-CS	0.84 (0.75–0.93)	94.2
Model 3—Baseline prognostic factors + CTP-CS	0.86 (0.77–0.94)	90.7

AIC: Akaike Information Criterion; CTA-CS: baseline single phase CTA-based collateral score; CTP-CS: baseline CTP-based collateral score. Higher C-statistic and lower AIC imply better models.

## Data Availability

Registry data can be made available on reasonable request via mrclean@erasmusmc.nl.
